# The Wide Potential Trophic Niche of the Asiatic Fruit Fly *Drosophila suzukii*: The Key of Its Invasion Success in Temperate Europe?

**DOI:** 10.1371/journal.pone.0142785

**Published:** 2015-11-18

**Authors:** Mathilde Poyet, Vincent Le Roux, Patricia Gibert, Antoine Meirland, Geneviève Prévost, Patrice Eslin, Olivier Chabrerie

**Affiliations:** 1 Unité Ecologie et Dynamiques des Systèmes Anthropisés (FRE-CNRS 3498), Université de Picardie Jules Verne, Amiens, France; 2 Laboratoire de Biométrie et Biologie Evolutive (UMR-CNRS 5558), Université Claude Bernard Lyon 1, Villeurbanne, France; 3 Groupe d'étude des milieux estuariens et littoraux (GEMEL) Picardie, Maison de l’Université de Picardie Jules Verne, Saint Valery-Sur-Somme, France; French National Institute for Agricultural Research (INRA), FRANCE

## Abstract

The Asiatic fruit fly *Drosophila suzukii* has recently invaded Europe and North and South America, causing severe damage to fruit production systems. Although agronomic host plants of that fly are now well documented, little is known about the suitability of wild and ornamental hosts in its exotic area. In order to study the potential trophic niche of *D*. *suzukii* with relation to fruit characteristics, fleshy fruits from 67 plant species were sampled in natural and anthropic ecosystems (forests, hedgerows, grasslands, coastal areas, gardens and urban areas) of the north of France and submitted to experimental infestations. A set of fruit traits (structure, colour, shape, skin texture, diameter and weight, phenology) potentially interacting with oviposition choices and development success of *D*. *suzukii* was measured. Almost half of the tested plant species belonging to 17 plant families allowed the full development of *D*. *suzukii*. This suggests that the extreme polyphagy of the fly and the very large reservoir of hosts producing fruits all year round ensure temporal continuity in resource availability and contribute to the persistence and the exceptional invasion success of *D*. *suzukii* in natural habitats and neighbouring cultivated systems. Nevertheless, this very plastic trophic niche is not systematically beneficial to the fly. Some of the tested plants attractive to *D*. *suzukii* gravid females stimulate oviposition but do not allow full larval development. Planted near sensitive crops, these “trap plants” may attract and lure *D*. *suzukii*, therefore contributing to the control of the invasive fly.

## Introduction

Biological invasions are considered as one of the major causes of biodiversity loss on the planet [1, 2]. Alien species may have serious impact on native communities, habitats, and ecosystem processes [3], therefore altering ecosystem services that might be key elements for human well-being [4–6]. In addition, if the life cycle of invasive species coincides with that of production systems, this may have a huge economic impact [7, 8]. Predicting the possible success of invasive exotic species together with acquiring an overall understanding of the rules applying to invasion ecology is very challenging considering the specificity of each invasion process. Indeed, the success of an invasion is linked to the history of the species’ introduction [9], each success depending on the probability of both climate and habitat matching (*i*.*e*. ecosystem invasibility) with the invader’s requirements [10, 11], and on the wide set of the species’ characteristics (i.e. the species’ invasiveness) [10, 12].

Unlike other phylogenetic groups, insects have long been neglected as to their potential ecological impact, except for those inducing sanitary or economic effects, especially when related to agriculture or forestry [13, 14]. About 1390 insect species have been introduced into Europe, including 98 Diptera originating mostly from North America and Asia, with phytophagous species being the most represented over the past decade [14]. Most of the Diptera species introduced are mainly associated with such anthropic environments as urban areas and agrosystems [15]. Among them, the *Drosophila* genus has experienced a long history of invasion (see the case of *D*. *melanogaster* [16]) due to the high fecundity and short generation time of its members, and to its great ability to adapt to changing environments [17, 18]. Recently, *Drosophila suzukii* [19] has become the 8^th^ species of this genus introduced into Europe and it is now by far the most damaging *Drosophila* species in agricultural areas [15, 20].

Currently, this pest is expanding rapidly in North and South America and in Europe [18, 21–23], with such a high velocity (about 1000 km per year) that the invasion is almost unprecedented [18]. *Drosophila suzukii* was first recorded in the south of France in 2009 and then dispersed to the north of the French territory during the following years [24], probably dispersing both on its own and via passive transport through fruit trade [18, 25, 26]. Dispersal is not a limiting factor to the expansion of this species [18] characterized by a migratory behaviour in its native and exotic habitats [18, 27]. The species is now largely established in natural and production-based systems in which it interacts with resident species and damages a large part of the fruit production [21, 24].

*Drosophila suzukii* is currently the object of intense research because of its huge impact on the small-fruit industry in Europe and North America where it was introduced [7, 28]. Unlike the vast majority of *Drosophila* flies whose trophic niches are based on fungi or rotten / over-ripened fallen fruits, the trophic niche of *D*. *suzukii* favours fresh and ripening fruits [24]. The fly is able to pierce fruit skin by using a serrated ovipositor enabling it to lay eggs deeply in the flesh of the fruit [29]. Fungi and bacteria develop on oviposition scars and gain access to the fleshy tissues of the fruit which will rot prematurely [28]. Commercial soft fruits, blueberries, strawberries, blackberries, raspberries, tomatoes, grapes, and fruit from fruit trees, cherries, kiwis, figs, apples, plums, peaches, among others [18, 22, 30–32], are suitable hosts potentially damaged by this fly. In 2008 the cost of *D*. *suzukii* invasion was estimated at 511 million dollars for all the crops in the USA [33]. In the south of France (the Dordogne region), strawberry growers lose an average of 5000 euros per farm per year [34]. As this economic impact results from the initial stages of a recent, still ongoing invasion, losses are likely to increase in the coming years leading to a serious reduction of the fruit producers’ income, up to 37% according to Goodhue *et al*. (2011) [7].

*Drosophila suzukii* is known to infest a wide variety of *Prunus* stone fruits in its native area [27, 35, 36] and numerous families of cultivated fruits in the agrosystems of its exotic range [17, 18, 30, 31, 37]. However, an extensive screening of wild and ornamental plants hosting *D*. *suzukii* in its exotic range, especially in temperate Europe, is still missing and the relative effects of fruit characteristics on host selection by *D*. *suzukii* are poorly known.

The plasticity of *D*. *suzukii* in its host preferences and nutritional requirements is one of the key of its success which may have led the fly to enlarge its fundamental and realized niche [38]. The fruits of different plant species are not equally suitable to the different life stages of *D*. *suzukii* (egg, larva, adult), as suitability depends on a large set of traits (volatile compounds, pH, shape, structure, firmness, quantity and quality of resources, colour…) either preventing, limiting or favouring the development of the fly [30, 31, 39]. Hence, analyzing relationships between the functional traits of the fruit and *D*. *suzukii* oviposition behaviour or larval development would help to understand the mechanisms underlying successful invasion [40] that are not explained by a taxonomic approach (*i*.*e*. species of different families can show similar fruit traits, while a great diversity of fruit shapes or colours can be found in a single family). In functional ecology, plant species and traits are grouped according to common responses to the environment (‘response traits’) or common effects on ecosystem processes (‘effect traits’) [41]. Fruit colour and shape are commonly considered as responses (‘response traits’) to fruit consumer behaviour-as with *D*. *suzukii*’s oviposition behaviour-, while fruit structure (including the presence of septa within complex fruits and internal partitions, as in *Rubus* polydrupes) and size/diameter will rather influence larval development by limiting resources (‘effect traits’). Therefore, fruit nutrients and weight may influence ecosystem processes through the quantity and quality of organic matter entering the trophic networks in the presence of *D*. *suzukii* populations.

In this study, we examine the relationships between the community of fleshy-fruited plants and the invader *D*. *suzukii* in a temperate region of Europe. More specifically, we addressed the following research questions: (1) how many plant species may host *D*. *suzukii* larvae and lead to the full development of imagos? (2) Do fruit traits (structure, colour, shape, skin texture, diameter and weight) affect *D*. *suzukii* oviposition choices and development success?

As *D*. *suzukii* is able to migrate across regions, along altitudinal and climatic gradients and within and between ecosystems according to the season [18, 27, 42], fruits of 67 wild and ornamental plants were collected over the course of an entire year in various ecosystems (forest, hedgerow, grassland, garden, coastal area…) of the north of France and exposed to infestation by *D*. *suzukii* under experimental conditions using no-choice tests.

## Materials and Methods

### Study area and field sampling

The study was carried out in the Picardy region, in the north of France (N 48°50'19''–50°21'59''; E 1°22'50''–4°15'23''; alt. 0–296 m). The climate is of the oceanic type, with a mean annual temperature of 10°C and an average annual rainfall of 700 mm. The geological substrate is mainly composed of Cretaceous chalk covered by clay and/or Quaternary loess. Sandy substrates and salt alluvium are locally found along the coast. Landscapes are diversified and consist of mosaics of openfields, bocages, forests, urban areas and coastal vegetation. All these types of landscape have been visited to identify the pool of fleshy-fruited plants that could be potentially used as host plants by *D*. *suzukii* over the four seasons of one year.

Between October 2011 and November 2012, fruits from 67 fleshy-fruited plant species (see details in Appendix A) were collected in forests, hedgerows, grasslands, coastal areas, gardens and urban areas in the Picardy region. This exhaustive species sampling covered the majority of fleshy-fruited plant species present in the north of France. Only 16 fleshy-fruited wild plant taxa of the north of France were not tested in the study: *Arum italicum* (exotic), *Convallaria maialis* (irregular fruiting in the forests of the north of France), *Cornus mas*, *Crataegus laevigata*, *Daphne laureola*, *Hypericum androsaemum*, *Lonicera periclymenum*, *Prunus laurocerasus* (irregular fruiting in the north of France), *Pyrus* sp., *Rosa arversis*, *Rubus caesius*, *R*. *ulmifolius*, *Sorbus torminalis*, *Tamus communis*, *Vaccinium myrtillus* (rarely fruiting in the forests of the north of France) and *Viburnum lantana*. For each plant species, fruits from five individuals separated by a minimum distance of 200 m were collected randomly and stored in individual paper bags. Fruits from each species of plant were collected only once at maturity during the season from all five individual plants. Every month, the presence of fruit of each species was recorded in the field. The fruits of 10 additional species (Appendix B) were collected but not included in the analyses because the number of fruit per individual or per species was too low to be tested. The colour of the fruit skin is commonly used to characterize their maturity in *D*. *suzukii* studies [24, 43, 44]. For instance, *Atropa belladonna* fruits turn from green (unripe) to black (ripe) before falling, while *Arum maculatum* fruits change from green to red at maturity [45]. All the fruits collected in this study were ripe, *i*.*e*. their skin had the colour typically associated with their maturity (*e*.*g*. entirely black for *Atropa belladonna* and entirely red for *Arum maculatum*), according to the indications of floras [45–48]. Plants were sampled on sites receiving no chemical treatments, excepted *Solanum tuberosum* (listed in the additional set of plant species of Appendix B, not included in the analyses) which was collected on a conventional agricultural field at the end of the production period. The sampling was conducted with the permission of the owners of private land and gardens. In public areas, no specific permissions were required (e.g. *Rubus fruticosus* is traditionally harvested in public forests for preparing jam). Field studies did not involve species currently known to be neither endangered nor protected, and many sampled species were ornamental plants commonly marketed in plant nurseries.

**Appendix A:** Characteristics of the 67 plant species tested in the study.

Num.	Species	Families (APG III)	Geographical origin	Used as ornamental or cultivated plant	Native (X = native in temperate Europe; (X) exotic in the north of France)	Exotic naturalized	Invasive	Collection site	Management of sampled individuals
1	*Aralia racemosa*	Araliaceae	North America	X				Garden	Planted
2	*Arum maculatum*	Araceae	North Europe		X			Forest	Spontaneous
3	*Asparagus officinalis*	Asparagaceae	Europe, Northern Africa, Western Asia	X	(X)			Garden—Crop edge	Planted—Spontaneous
4	*Atropa belladonna*	Solanaceae	Europe, North Africa, Western Asia		X			Garden—Forest edge	Spontaneous
5	*Aucuba japonica*	Garryaceae	Asia	X				Urban park	Planted
6	*Berberis julianae*	Berberidaceae	Asia	X				Hedgerow	Planted
7	*Berberis thunbergii*	Berberidaceae	Asia	X				Hedgerow	Planted
8	*Bryonia dioica*	Cucurbitaceae	Central and Southern Europe		X			Hedgerow	Spontaneous
9	*Callicarpa bodinieri*	Lamiaceae	West and Central Asia	X				Garden	Planted
10	*Cornus sanguinea*	Cornaceae	Europe and West Asia		X			Hedgerow	Spontaneous
11	*Cornus sericea*	Cornaceae	Northern and Western North America	X		X	X	Hedgerow	Planted
12	*Cotoneaster horizontalis*	Rosaceae	China	X		X		Green area	Planted
13	*Cotoneaster salicifolius*	Rosaceae	China	X		X		Garden	Planted
14	*Crataegus monogyna*	Rosaceae	Europe, Northwest Africa and Western Asia	X	X			Hedgerow	Spontaneous
15	*Elaeagnus* x *ebbingei*	Elaeagnaceae	Europe North America	X				Hedgerow	Planted
16	*Euonymus europaeus*	Celastraceae	Europe		X			Forest—Hedgerow	Spontaneous
17	*Fragaria vesca*	Rosaceae	Northern Hemisphere		X			Forest edge	Spontaneous
18	*Frangula alnus*	Rhamnaceae	Europe, Northernmost Africa, Western Asia		X			Wetland—Park	Spontaneous
19	*Gaultheria procumbens*	Ericaceae	North America	X				Garden	Planted
20	*Hedera helix*	Araliaceae	Europe and Western Asia		X			Hedgerow	Spontaneous
21	*Hippophae rhamnoides* subsp. *rhamnoides*	Elaeagnaceae	Europe and Asia	X	X			Coastal area—Garden	Spontaneous
22	*Ilex aquifolium*	Aquifoliaceae	Western and Southern Europe, Northwest Africa, Southwest Asia	X	X			Forest	Spontaneous
23	*Juniperus communis*	Cupressaceae	Northern Hemisphere	X	X			Garden	Planted—Spontaneous
24	*Ligustrum ovalifolium*	Oleaceae	Japan	X				Hedgerow—Garden	Planted
25	*Ligustrum vulgare*	Oleaceae	Europe, Asia, North Africa		X			Hedgerow	Spontaneous
26	*Lonicera caprifolium*	Caprifoliaceae	Europe	X	(X)			Park	Planted—Spontaneous
27	*Lonicera nitida*	Caprifoliaceae	China	X		X		Hedgerow—Garden	Planted
28	*Lonicera xylosteum*	Caprifoliaceae	Europe, Asia	X	X			Grassland—Hedgerow	Spontaneous
29	*Mahonia aquifolium*	Berberidaceae	Western North America	X		X	X	Green area	Planted
30	*Mahonia x media*	Berberidaceae	Northern Ireland	X				Green area	Planted
31	*Mespilus germanica*	Rosaceae	Europe, Asia	X	X			Forested park	Spontaneous
32	*Morus* sp.	Moraceae	Asia	X				Green area	Planted
33	*Paris quadrifolia*	Melanthiaceae	Europe		X			Forest	Spontaneous
34	*Parthenocissus inserta*	Vitaceae	North America	X		X	X	Hedgerow	Spontaneous
35	*Physalis alkekengi*	Solanaceae	Europe, Asia	X				Garden	Planted
36	*Phytolacca americana*	Phytolaccaceae	North America	X		X	X	Garden	Planted—Spontaneous
37	*Polygonatum multiflorum*	Asparagaceae	Europe, Asia		X			Forest	Spontaneous
38	*Prunus avium*	Rosaceae	Europe, Asia	X	X			Hedgerow	Planted—Spontaneous
39	*Prunus lusitanica*	Rosaceae	South Europe	X				Hedgerow	Planted
40	*Prunus mahaleb*	Rosaceae	Southern Europe, Asia		X			Grassland	Spontaneous
41	*Prunus padus*	Rosaceae	Northern Europe, Northern Asia	X	X			Garden—Forest edge	Planted—Spontaneous
42	*Prunus serotina*	Rosaceae	North America	X		X	X	Forest	Spontaneous
43	*Prunus spinosa*	Rosaceae	Europe, Western Asia, Northwest Africa	X	X			Hedgerow	Spontaneous
44	*Pyracantha coccinea*	Rosaceae	Southeast Europe, East to Southeast Asia	X				Hedgerow	Planted
45	*Pyrus calleryana*	Rosaceae	Asia	X				Urban area	Planted
46	*Rhamnus cathartica*	Rhamnaceae	Europe, Northwest Africa, Western Asia		X			Hedgerow	Spontaneous
47	*Ribes nigrum*	Grossulariaceae	Europe, Northern Asia	X	X			Park	Planted
48	*Ribes rubrum*	Grossulariaceae	Europe	X	X			Forest	Spontaneous
49	*Ribes sanguineum*	Grossulariaceae	North America	X		X		Hedgerow	Planted
50	*Rosa canina*	Rosaceae	Europe, Northwest Africa, Western Asia.		X			Hedgerow—Coppice	Spontaneous
51	*Rubia tinctorum*	Rubiaceae	Europe, Asia	X	(X)			Garden	Planted
52	*Rubus fruticosus* agg.	Rosaceae	Northern Hemisphere		X			Hedgerow	Spontaneous
53	*Rubus idaeus*	Rosaceae	Northern Hemisphere		X			Park	Planted—Spontaneous
54	*Ruscus aculeatus*	Asparagaceae	Europe, Asia	X	X			Park—Forest	Planted—Spontaneous
55	*Sambucus ebulus*	Adoxaceae	Europe, Asia	X	(X)			Garden	Planted
56	*Sambucus nigra*	Adoxaceae	Europe		X			Hedgerow—forest edge	Spontaneous
57	*Solanum dulcamara*	Solanaceae	Europe, Asia		X			Garden	Spontaneous
58	*Solanum dulcamara* f. *littorale*	Solanaceae	Europe, Asia		X			Coastal garden, town	Spontaneous
59	*Solanum nigrum*	Solanaceae	Europe, Asia		X			Garden	Spontaneous
60	*Sorbus aria*	Rosaceae	Europe	X	X			Park	Planted
61	*Sorbus aucuparia*	Rosaceae	Europe, Asia	X	X			Green area	Planted—Spontaneous
62	*Symphoricarpos albus*	Caprifoliaceae	North America	X		X		Park	Planted
63	*Symphoricarpos* x *chenaultii*	Caprifoliaceae	America	X		X		Park	Planted
64	*Taxus baccata*	Taxaceae	Europe, Northwest Africa, Southwest Asia	X	(X)			Park	Planted
65	*Viburnum opulus*	Adoxaceae	Europe, Asia	X	X			Hedgerow—garden	Planted
66	*Viburnum tinus*	Adoxaceae	South Europe, North Africa	X				Park—Garden	Planted
67	*Viscum album*	Santalaceae	Europe, Western and Southern Asia		X			Apple tree in grassland	Spontaneous

**Appendix B:** Emergence of *Drosophila suzukii* imagos from the fruits of an additional set of plant species. These additional species were collected but not included in the analyses because the number of fruit collected per individual or per species was too low to be tested.

Plant species	Number of sampled individuals	Number of sampled fruits	Mean number of *D*. *suzukii* eggs per fruit	± S.E	Mean number of emerging adults of *D*. *suzukii* per fruit
*Cotoneaster bullatus*	1	10	2.80	0.05	0.00
*Cotoneaster watereri*	1	10	2.90	0.09	0.00
*Duchesnea indica*	2	20	0.20	0.01	0.00
*Fuchsia* sp.	1	10	1.20	0.05	1.20
*Iris* sp.	1	10	0.00	0.00	0.00
*Malus sylvestris*	3	24	0.00	0.00	0.00
*Rubia peregrina*	1	10	0.00	0.00	0.00
*Skimmia japonica*	1	10	0.00	0.00	0.00
*Solanum tuberosum*	2	20	0.00	0.00	0.00
*Vaccinium uliginosum*	1	10	1.20	0.04	0.20

### Allotments of fruit and laboratory tests

For each plant species, a total of 150 fruits (1 species x 5 individuals x 3 tests x 10 fruits) was used in laboratory tests. The fruits were tested immediately after sampling. For each individual of each species, 30 fruits were randomly selected. The fruits were carefully examined with a stereomicroscope (Leica M 165C) and those already damaged or attacked by animals or pathogens were excluded. The 30 fruits were split into three sets of ten fruits and placed in ventilated transparent plastic boxes (15 cm x 10 cm x 5cm) to perform three types of tests.

In a first test, subsequently termed ‘adult emergence test’, 10 fruits were exposed to 3 *D*. *suzukii* mated females for 24 hours following the protocol set up by Poyet *et al*. (2014) [24]. After 24 hours, the number of eggs laid in each fruit was counted under a Leica M 165C stereomicroscope. The presence of eggs oviposited in fruits was identified by the holes drilled by the females’ ovipositor and by the presence of egg filaments [24]. During those experiments, every hole observed contained one single *D*. *suzukii* egg. The number of *D*. *suzukii* flies emerging from each test sample was checked daily for two months, the flies were counted then removed from the experimental boxes to avoid new oviposition. In the second test (‘larvae development test’), 10 fruits were exposed to 3 *D*. *suzukii* females for 24 hours. After 24 hours, the number of eggs laid in each fruit was counted. After one week, the fruits were dissected and the total number of larvae that were present in and on the fruits and in the test plastic box was recorded. In the third test, a set of 10 fruits was used as a control to monitor the potential fruit contaminations by other insects.

In all the tests, the strain of *D*. *suzukii* collected in 2011 in the forest of Compiègne, in Picardy [24, 49], was used and maintained under an LD 13: 11 h photocycle at 20°C. The *D*. *suzukii* strain was mass reared and fed with a regular banana Drosophila diet [50]. Five-days-old mated females were used for each experiment to prevent the delay of eggs laying in young females.

### Fruit traits

We targeted fruit characteristics associated with three fundamental stages of the *D*. *suzukii* life cycle, *i*.*e*. egg laying, larval development and adult emergence. A set of five biological traits (including a total of 16 trait categories described hereafter) commonly related to oviposition choice and larva survival [51–55] was retained: maximum fruit diameter (fruit weight was also measured, but was redundant with fruit diameter and not retained in the final analyses; see explanations in the next paragraph), type (i.e. structure, defined hereafter), colour, shape and skin texture. Information was collected or extracted from flora [45–48] and plant databases [56, 57]. Fruit weight and maximum diameter were individually measured before laboratory tests on the 6694 fruits used for larval development and adult emergence experiments, and treated as continuous variables. Fruit type, colour, shape and skin were treated as categorical variables and the optimal number of categories was defined so as to be ecologically meaningful and have balanced sizes [58, 59]. Fruit type included four categories (1: berry; 2: drupe; 3: polydrupe and pseudo-polydrupe; 4: other fruit with complex structures: pseudo-fruit, complex fruit, multilocular capsule, aril); fruit colour at maturity included six categories (1: black; 2: red; 3: pink; 4: orange/light brown; 5: white; 6: blue); fruit shape included two categories (1: spherical; 2: oval) and fruit skin texture, three categories (1: smooth and waxy, glossy, shiny; 2: smooth and pruinose; 3: rough, irregular). The plant species nomenclature follows Lambinon *et al*. (2004) [48] and plant families follow the APG III phylogeny [60].

### Data analyses

The effects of fruit traits on the number of *D*. *suzukii* eggs, larvae and adults emerging from fruits were examined using generalized linear models (GLM) with a Poisson distribution and a log-link term [61]. In the egg-model (*n* = 67 plant species tested), the number of *D*. *suzukii* eggs was the response variable, the fruit diameter was used as a fixed covariate, and the fruit type, colour, shape, surface type and skin thickness were used as fixed factors. In the larva-model, the number of *D*. *suzukii* larvae (*n* = 60 plant species tested) was the response variable, the fruit diameter was used as a fixed covariate, and the fruit type was used as a fixed factor. Seven species were excluded from this analysis (*Berberis julianae*, *Gaultheria procumbens*, *Ligustrum vulgare*, *Lonicera caprifolium*, *Mespilus germanica*, *Pyrus calleryana* 'Chanticleer', *Sorbus aria*) because too few undamaged fruits per individual plant were available to be tested. In the adult-model (*n* = 67 plant species tested), the number of *D*. *suzukii* adults emerging from fruits was the response variable, the fruit diameter was used as a fixed covariate, and the fruit type was used as a fixed factor. To fulfil the normality assumption, fruit diameter and weight were log_10_-transformed. As fruit diameter and weight were highly correlated (R = +0.813; p<0.0001), fruit weight (less correlated with the number of eggs laid in the fruits by the flies than the diameter was) was excluded from the analyses to avoid redundant explanatory variables in the models. SPSS v. 17.0 (IBM Corp., Somers, NY, USA) was used in all the analyses.

## Results

**Fig 1:**
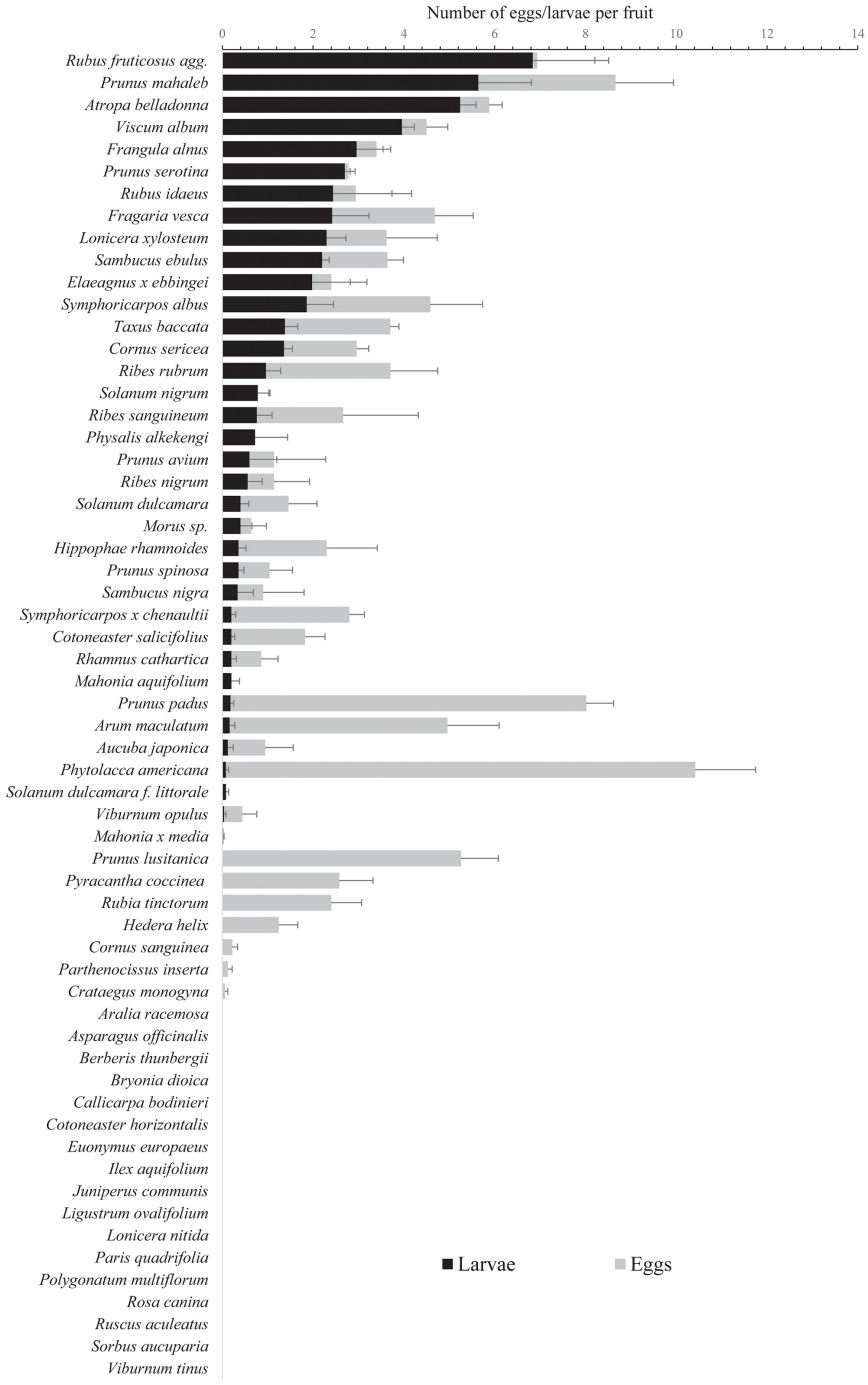
Mean number (± S.E.) of *Drosophila suzukii* eggs and larvae per fruit in the ‘larvae development test’.

**Fig 2:**
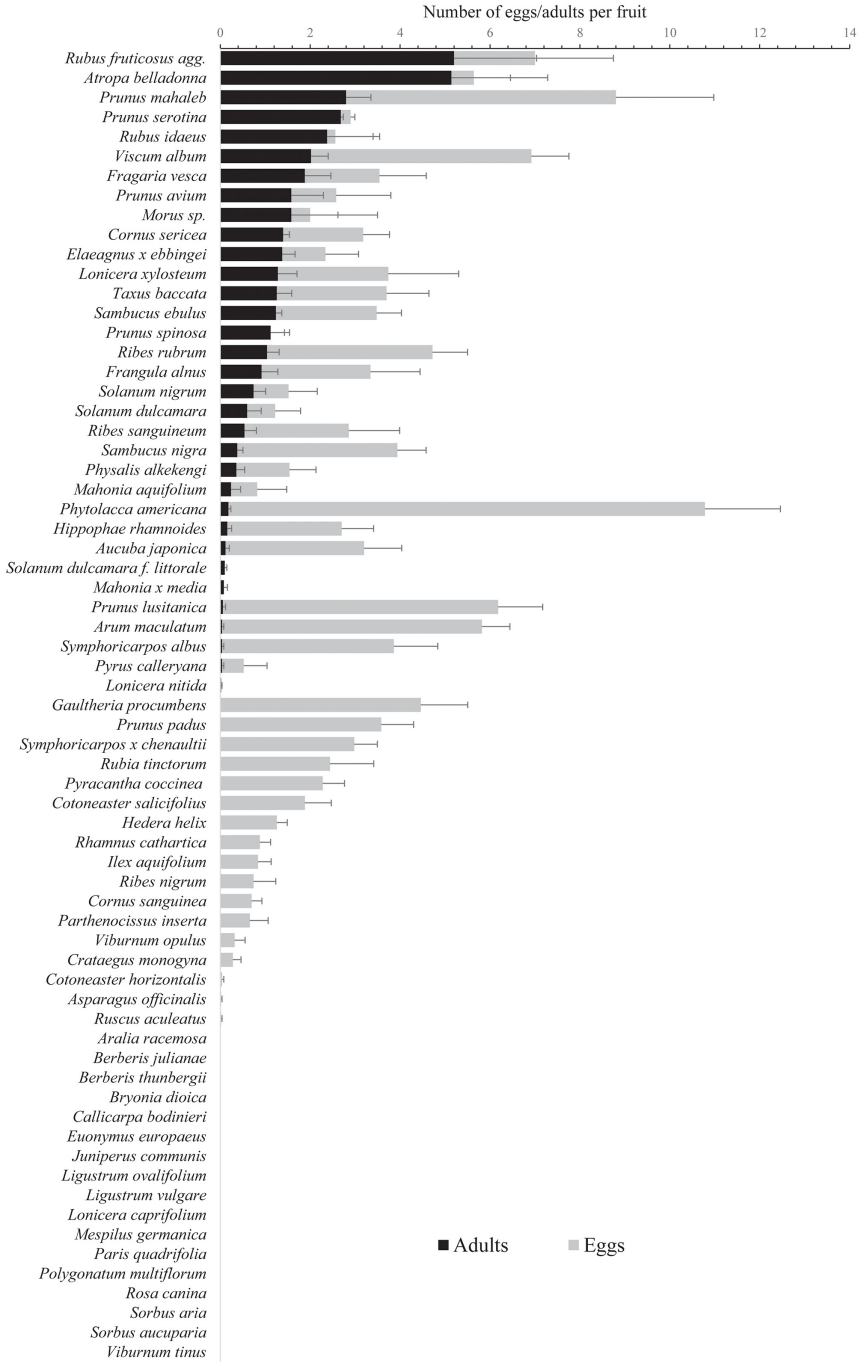
Mean number (± S.E.) of *Drosophila suzukii* eggs and imagos per fruit in the ‘adult emergence tests’.

A very wide range of plant families was used by *D*. *suzukii* females and allowed adult emergence: 2 *Adoxaceae*, 1 *Aracea*, 2 *Berberidaceae*, 3 *Caprifoliaceae*, 1 *Cornacea*, 2 *Elaeagnacea*, 1 *Garryacea*, 2 *Grossulariaceae*, 1 *Moracea*, 1 *Phytolaccacea*, 1 *Rhamnacea*, 9 *Rosaceae*, 1 *Santalacea*, 5 *Solanaceae*, 1 *Taxacea* (plus 1 *Onagracea* and 1 *Ericacea* when considering the additional set of tested species in Appendix B). The plant species showing the highest number of *D*. *suzukii* eggs per fruit were *Phytolacca americana*, *Prunus mahaleb*, *Rubus fruticosus* agg., *Viscum album* and *Prunus lusitanica* (Figs 1 and 2); those hosting the highest number of larvae were *Rubus fruticosus* agg., *Prunus mahaleb*, *Atropa belladonna*, *Viscum album* and *Frangula alnus*, and those leading to the highest number of imago emergences were *Rubus fruticosus*, *Atropa belladonna*, *Prunus mahaleb*, *Prunus serotina*, and *Rubus idaeus* (Fig 2). The highest mean number of eggs per fruit was 10.78 (± 1.68) for *Phytolacca americana*, the highest mean number of larvae per fruit was 6.84 (± 1.37) for *Rubus fruticosus* agg., and the highest mean number of adult emergences per fruit was 5.20 (± 1.83) for *Rubus fruticosus* agg. The developmental times of *D*. *suzukii* from eggs to adults among the tested fruits were reported in Appendix C. The developmental time was negatively correlated with the number of imago emergences per fruit (R = -0.512; p = 0.002) and not significantly correlated with neither the fruit diameter (R = -0.173; p = 0.335) or weight (R = -0.210; p = 0.240).

Among the 67 plant species tested, 33 (49.25%) allowed the emergence of *D*. *suzukii* imagos, 6 (8.96%) hosted larvae that did not reach the adult stage, 11 (16.42%) hosted eggs that did not hatch, and 17 (25.37%) did not host any eggs (Figs 1 and 2).

**Appendix C:** Mean (± S.E.) developmental time (in days) of *Drosophila suzukii* from eggs to adults among the tested fruits.

Plant species	Mean	S.E.
*Rubus fruticosus* agg.	15.00	0.00
*Sambucus nigra*	15.00	0.00
*Morus* sp.	15.14	0.24
*Atropa belladonna*	16.93	0.07
*Rubus idaeus*	17.76	0.12
*Prunus avium*	18.14	0.07
*Solanum dulcamara*	18.63	0.21
*Solanum nigrum*	18.92	0.24
*Frangula alnus*	19.30	0.25
*Lonicera xylosteum*	19.66	0.24
*Prunus mahaleb*	19.75	0.20
*Mahonia aquifolium*	19.83	0.51
*Viscum album*	19.94	0.11
*Mahonia* x *media*	20.00	0.00
*Fragaria vesca*	20.10	0.22
*Ribes rubrum*	20.10	0.26
*Physalis alkekengi*	20.67	0.60
*Prunus lusitanica*	21.00	0.00
*Solanum dulcamara* f. *littorale*	21.00	0.00
*Symphoricarpos albus*	21.00	0.00
*Elaeagnus* x *ebbingei*	21.07	0.16
*Ribes sanguineum*	21.22	0.15
*Prunus serotina*	21.54	0.20
*Taxus baccata*	21.97	0.16
*Arum maculatum*	22.00	0.00
*Sambucus ebulus*	22.53	0.15
*Cornus sericea*	22.81	0.06
*Aucuba japonica*	23.00	0.00
*Phytolacca americana*	23.67	1.08
*Prunus spinosa*	23.82	0.39
*Pyrus calleryana*	24.00	0.00
*Hippophae rhamnoides*	24.63	0.18
*Lonicera nitida*	27.00	0.00

### Effects of fruit traits

The GLM showed significant effects of fruit traits on the number of *D*. *suzukii* eggs, larvae, and adults emerging from fruits (Table 1). The number of eggs, larvae and adults increased significantly with the fruit diameter. The number of eggs was higher in berries and drupes than in the other fruit types, higher in white or black fruits than in fruits of other colours, higher in oval fruits than in spherical ones and higher in rough fruits than in smooth ones, especially in those with a pruinose coating. The number of larvae and emerging adults was higher in polydrupes than in the other types of fruit and was also higher in drupes and berries than in fruits with a complex structure.

**Table 1:** Generalized linear models showing the effects of fruit traits on the number of *D*. *suzukii* eggs, larvae and adults emerging per 100 fruits.

Response variables	Explanatory variables	Category	Par. est. *β* ^1^	S.E.^2^	Wald X^2^	D.F.^3^	*P*-value
**Number of eggs (*n* = 67 plant species)**	**Intercept**		5.136	0.063	6602.11	1	<0.001
	**Fruit diameter (log10)**		2.181	0.058	1424.40	1	<0.001
	**Fruit type**				1370.94	3	<0.001
		**Berry**	1.399	0.039	1297.88	1	<0.001
		**Drupe**	1.357	0.042	1033.24	1	<0.001
		**Polydrupe**	0.816	0.053	233.30	1	<0.001
		**Other (complex structure)**	0	-	-	-	-
	**Fruit color**				2523.05	5	<0.001
		**Black**	1.291	0.050	675.12	1	<0.001
		**Red**	0.505	0.051	97.72	1	<0.001
		**Pink**	0.354	0.060	35.10	1	<0.001
		**Orange-light brown**	-0.791	0.070	128.77	1	<0.001
		**White**	1.503	0.056	718.09	1	<0.001
		**Blue**	0	-	-	-	-
	**Fruit shape**				475.66	1	<0.001
		**Spherical**	-0.572	0.026	475.66	1	<0.001
		**Oval**	0	-	-	-	-
	**Fruit skin**				1601.04	2	<0.001
		**Smooth, waxy, shiny**	-1.171	0.041	817.62	1	<0.001
		**Smooth, pruinose**	-2.174	0.054	1600.22	1	<0.001
		**rough**	0	-	-	-	-
**Number of larvae (*n* = 60 plant species)**	**Intercept**		3.929	0.050	6170.05	1	<0.001
	**Fruit diameter (log10)**		3.746	0.105	1263.09	1	<0.001
	**Fruit type**				633.96	3	<0.001
		**Berry**	0.653	0.055	142.71	1	<0.001
		**Drupe**	0.846	0.056	228.98	1	<0.001
		**Polydrupe**	1.446	0.061	561.24	1	<0.001
		**Other (complex structure)**	0	-	-	-	-
**Number of adults (*n* = 67 plant species)**	**Intercept**		2.996	0.059	2618.24	1	<0.001
	**Fruit diameter (log10)**		4.029	0.115	1226.80	1	<0.001
	**Fruit type**				1369.83	3	<0.001
		**Berry**	1.239	0.066	356.70	1	<0.001
		**Drupe**	1.376	0.067	419.30	1	<0.001
		**Polydrupe**	2.291	0.065	1236.05	1	<0.001
		**Other (complex structure)**	0	-	-	-	-

^1^: parameter estimate

^2^: standard error

^3^: degrees of freedom.

### Range of plant species and fruit phenology

Among the 33 plant species that allowed the emergence of *D*. *suzukii* adults, 21 (63.6%) were ornamental or cultivated species, 14 (42.4%) were exotic, and 4 species (12.1%) were invasive in the region. Several naturalized plant species (*i*.*e*. observed in nature but not considered as invasive at present: *Lonicera nitida*, *Ribes sanguineum* and *Symphoricarpos albus*) also allowed the emergence of *D*. *suzukii* adults (Appendix A and Fig 2). Among the 67 plants tested, the number of larvae per fruit was lower in the ornamental plant species (0.49 ± 0.12 larvae.fruit^-1^) than in the others (1.57 ± 0.50 larvae.fruit^-1^; *t* = -2.799, *p* = 0.007). The number of emerging adults per fruit was lower too, in the ornamental plant species (0.36 ± 0.09 larvae.fruit^-1^) than in the others (1.06 ± 0.35 larvae.fruit^-1^; *t* = -2.548, *p* = 0.013).

**Fig 3:**
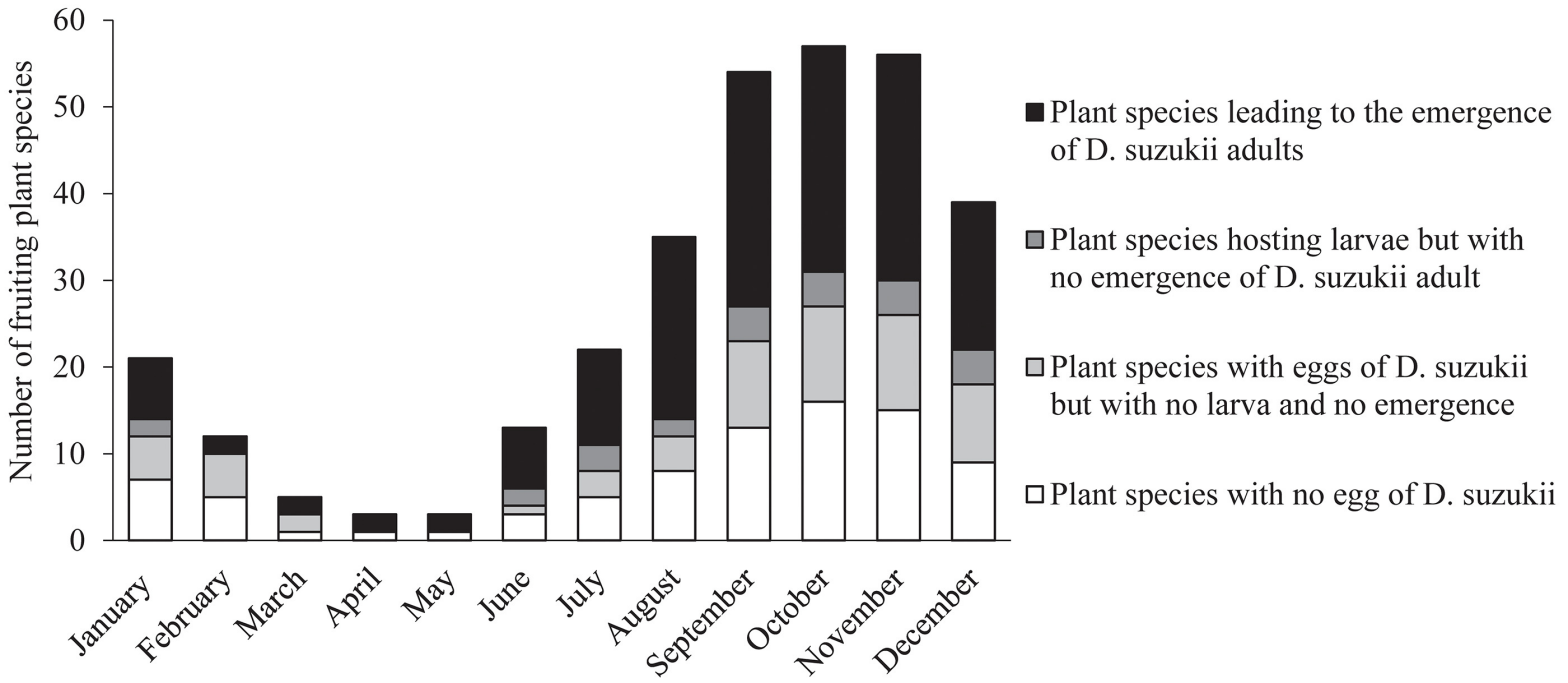
Fruiting periods of studied plant species. Plant species are grouped into four categories according to their suitability for the different development stages of *Drosophila suzukii*.

**Fig 4:**
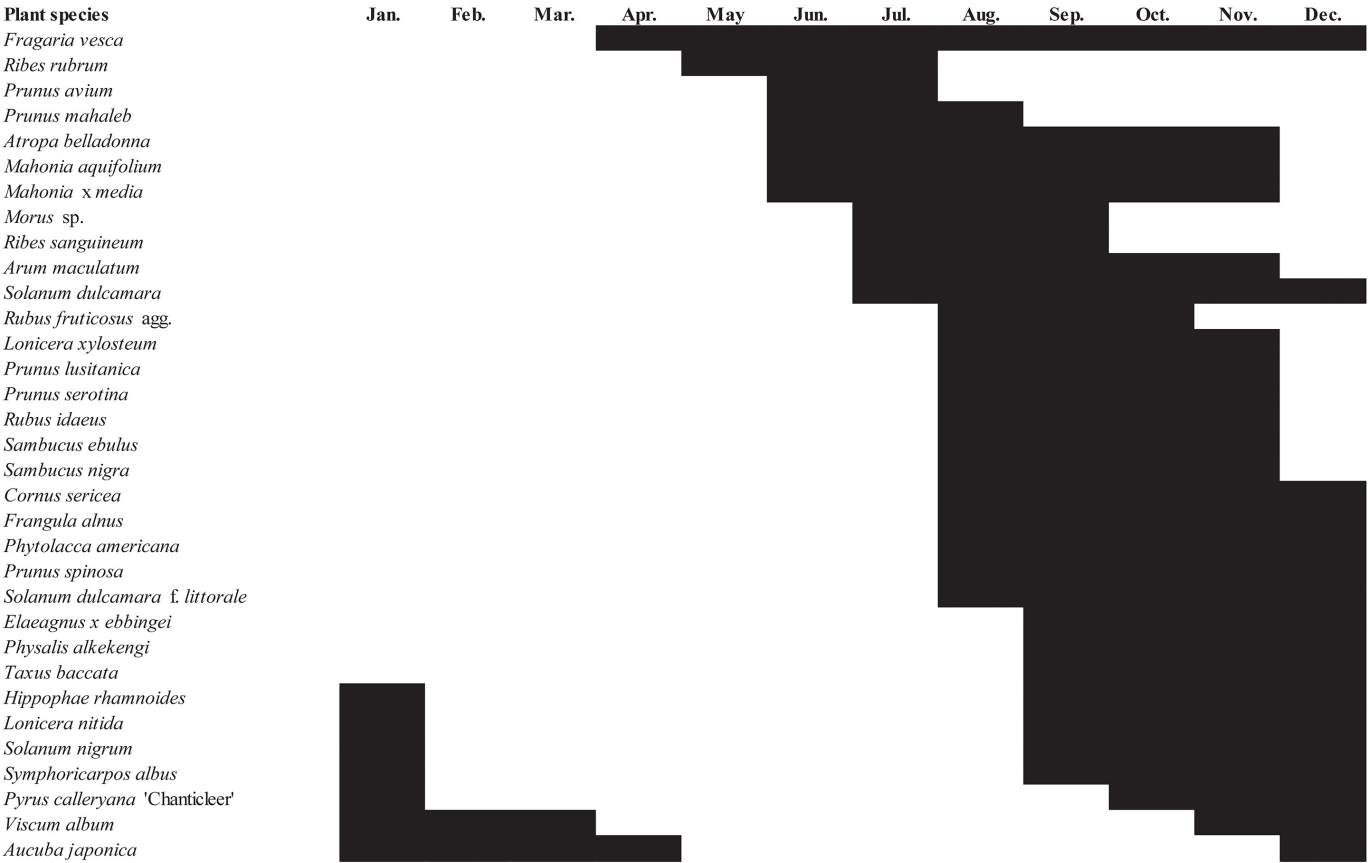
The fruit seasonality (recorded in the sampling sites in Picardy in 2011–2012) of the plant species that successfully hosted *Drosophila suzukii*.

The number of plant species with fruits potentially suitable for the development of *D*. *suzukii* offspring was high between September and December with a peak in October (Fig 3). Only a few host plants suitable for *D*. *suzukii* were fruiting in early spring. However, the fruits of several potential native and exotic hosts remained hanging on plants during the winter (*Viscum album*, *Aucuba japonica*, *Hippophae rhamnoides* subsp. *rhamnoides*, *Symphoricarpos albus*). Fig 4 showed that host plants suitable for *D*. *suzukii* were potentially available across the four seasons. *Viscum album* and *Aucuba japonica* were the only suitable plant species in February and March and represented a resource providing continuity between winter and spring. These 33 suitable host plants were found in the different habitats investigated in the region: forest (*n* = 4 species), hedgerow (*n* = 9), grassland (*n* = 3), wetland (*n* = 1), coastal areas (*n* = 2), garden (*n* = 6), urban area and park (*n* = 8).

## Discussion

### Polyphagy of *D*. *suzukii*

Almost half of the 67 plant species tested in the study allowed the emergence of *D*. *suzukii* imagos and a total of 17 plant families, *i*.*e*. 56.7% of the 30 families tested (including the additional families of Appendix B), also enabled the development of *D*. *suzukii* adults. These findings, *i*.*e*. the extreme polyphagy of *D*. *suzukii* in terms of plant species and families, may help explain the exceptional success of this fly in matters of invasion, both locally and across the globe.

Firstly, the extremely high number of host plant species suitable for the development of *D*. *suzukii* indicates that the fly benefits from a large amount and diversity of resources that may have efficiently contributed to the success of its invasion. This wide polyphagy was mainly reported on agronomic varieties of cultivated fruits [31, 39] and gives *D*. *suzukii* several advantages. A multi-fruit diet reduces the time spent searching for food, limits the effect of resource stochasticity within and between years, increases enemy-free space, provides the fly with nutrient complementation, or attenuates the possible toxic effects of some fruits [62–64]. Moreover, a mixed diet may also increase the survival rate and the fecundity of adult females [64]. The large pool of host plants potentially used by *D*. *suzukii* in Picardy is widely distributed among both natural ecosystems and gardens throughout Europe. Among the suitable hosts detected in the present study are plants that *D*. *suzukii* probably encountered on its historical invasion roads from the south to the north of Europe [17, 65]. Indeed, some host plants are typical of the Mediterranean region where *D*. *suzukii* was first reported in Europe (for instance, *Prunus lusitanica* which is endemic in the Iberian Peninsula and in north-west Africa is used as an ornamental plant in France) [21]. These host plants are commonly found in the temperate forests and hedgerows of Western and Central Europe (*Arum maculatum*, *Fragaria vesca*, *Prunus avium*, *Prunus spinosa*, *Rubus idaeus*, *R*. *fruticosus* agg., *Sambucus nigra*…), or are characteristic of cold, mountainous areas, and of the north of Europe (*Vaccinium uliginosum*). Therefore, a further altitudinal and latitudinal expansion of *D*. *suzukii* area can be expected, as suitable fleshy-fruited plants are already present in cold areas and as the fly can migrate towards mountains, or overwinter [18, 27, 66].

Secondly, the host’s phylogeny is not a barrier to infestation by *D*. *suzukii*. Indeed, host families are phylogenetically very different and extremely distant on the APG III classification tree [60] as they belong to different Orders, Classes and Divisions: for example, *D*. *suzukii* successfully develops in the fruits of *Taxus baccata* (Family: *Taxacea*; Order: *Pinale*; Class: *Pinopsida*), *Arum maculatum* (Fam.: *Aracea*; Ord.: *Alismatale*; Cl.: *Liliopsida*), *Mahonia aquifolium* (Fam.: *Berberidacea*; Ord.: *Ranunculale*; Cl.: *Liliopsida*) and *Sambucus nigra* (Fam.: *Adoxaxea*; Ord.: *Dipsacale*; Cl.: *Liliopsida*). Many plant families include both kinds of species, either resistant or sensitive to *D*. *suzukii*. This suggests that the fruit’s traits matter more than the plants’ evolutionary history. A broader analysis of the hosts’ phylogeny across the different continents invaded by the fly could help understand the influence of preference- and performance-related traits of *D*. *suzukii* on host range [67]. Among the plant families infested by *D*. *suzukii*, many are known to produce toxic secondary compounds [68]. For example, alkaloids that are specific to each taxonomic group of plants and neurotoxic to mammals [68], have been used as deterrents to larvae or biocides to control insect pests [69–76]. *Solanaceae* are one of the plant families that produces the largest variety of toxic molecules, including alkaloids, concentrated in the fruits [68, 76]. Surprisingly, among the Solanacea family, *Atropa belladonna* is one of the best hosts for *D*. *suzukii* (Figs 1 and 2) while it predominantly contains tropane alkaloids [77–79], in addition to cuscohygrine, apotropine, belladonine and scopoline [80–83]. *Solanum dulcamara* and *S*. *nigrum* also allow the emergence of imagos (Fig 2) though harbouring numerous alkaloids [76, 84–87] that may act as feeding deterrents for insect larvae [70]. *D*. *suzukii* also successfully develops in a large set of other tested fruits (Fig 2) characterized by the presence of alkaloids or other toxic compounds such as glycosides, terpenoids and phenylpropanoids [88]. The high number of toxic plants permitting *D*. *suzukii* development suggests that larvae may possess a substantial set of enzymes enabling them to process these secondary compounds. Enzymatic detoxification ability has already been reported in polyphagous insects [89] and in particular in *Drosophila melanogaster* which shows a resistance to the alkaloids produced by the cacti on which larvae and adults feed [90]. *D*. *melanogaster* thus evades competition with other *Drosophila* species unable to use this toxic resource [91]. Feeding on toxic plants may confer several other advantages to the invasive fly. Numerous insects, especially at larval stages, can store in their tissues high quantities of alkaloids and other toxins present in their diet, thus increasing their resistance to pathogens [92] and parasites [93], or avoiding attacks by predators such as birds [94, 95]. The consumption of toxins by the *Drosophila* species can also be a strategy of medication against parasites [93, 96], which lays down a valuable hypothesis explaining why toxic fruit can be beneficial to *D*. *suzukii*.

The wide polyphagy of *D*. *suzukii* contrasts with the diet of other invasive insect pests in France and Europe—monophagous or oligophagous species- like the grapevine phylloxera (*Daktulosphaira vitifoliae*) and the Colorado potato beetle (*Leptinotarsa decemlineata*) [97, 98], two historical and emblematic pests on the continent. The oligophagy among insect pests facilitates their control, as they do not benefit by alternative food resources when their main host plant has become resistant to infestation. For example, the damages caused by *Daktulosphaira vitifoliae* and its survival rate are dramatically reduced by using plants naturally or artificially resistant to phylloxera as rootstocks [99]. This type of pest control by host resistance would be difficult to use against the recent invasions of polyphagous pests like *D*. *suzukii*, *Halyomorpha halys* [100, 101], *Bemisia tabaci* [102] or *Popillia japonica* [103], since they all have a large set of host plants in their exotic range. For these new multi-host pests, other integrated and ecological control strategies need to be developed, including the management of their natural reservoirs in the vicinity of crops and that of crop diversity. Although host diversity is generally beneficial to polyphagous insects, in the presence of a mixture of host plants these insects may experience difficulties in matters of decision-making when selecting food and oviposition sites [102]. This behavioural disturbance may reduce their performance. For example, increasing the host plant diversity in the environment of multi-host pests may lead individuals to move more, to switch between plants more frequently, and to lose energy by feeding in each place for short periods of time [102, 104]. Unlike *D*. *suzukii*, other invasive *Drosophila* species (for example, *D*. *subobscura* in America [105], *D*. *melanogaster* in Australia [106], or *D*. *simulans* in Europe [107]) are mainly used as model species for genetic studies but are not considered to be important pests because they mainly feed on diversified but rotten substrates and do not damage fruit and vegetable productions.

The controlled environment in our experiments provided the homogeneous conditions necessary to perform sample comparisons and to avoid the biases caused by the environmental variability commonly observed in natural infestations (depending on climate or other stochastic factors in the field). The findings recorded here do not necessarily describe overall fruit infestation in natural environments but they do point to a potential trophic niche that can be partially occupied by the fly according to local factors. Moreover, fruit maturity may also influence the rate of infestation in the field as well as in laboratory conditions [24, 43]. Indeed, *D*. *suzukii* lays more eggs on ripe fruits for some plant species [43], while for other host plants more eggs are found on ripening fruits [24]. The influence of fruit maturation on the fly’s behavioural preferences still remains debated as a recent study showed that the developmental stage of fruit alone does not explain the ecological niche observed for *D*. *suzukii* [108], and that other plant traits need to be examined to understand fly-fruit interactions.

### The role of fruit traits in *D*. *suzukii* infestation

All along their evolutionary history, Angiosperms developed a wide range of fruit traits that protect them against predators or facilitate the activity of frugivorous insects [89, 109, 110]. To continue to exist in the environment, *D*. *suzukii* needs to ensure the success of the fundamental steps of its relationship with fruits: oviposition, development, and adult emergence.

#### Fruit size

The first important fruit trait associated with the success of the different *D*. *suzukii* life stages is fruit size, which is measured by the fruit’s diameter and weight (Table 1). Larger fruits increase the number of *D*. *suzukii* adults emerging from the fruits, as previously reported with *Prunus serotina* [24]. The correlation between fruit size and egg-clutch size is proved for many plant-insect associations [53, 111] but the fruit size can also be even more important than the fruit type itself [53] as it may indicate to gravid females what amount of resources is available for their progeny. Fruit diameter is also correlated to the number of developing larvae and emerging imagos (Table 1), but the developmental time was negatively correlated with the number of imago emergences. Indeed, multiple infestations of small fruits may prove lethal to competing larvae whilst pupal mass is known to increase with fruit size [112]. Our results corroborate those of Dukas *et al*. (2001), showing that, in small fruit, competition between larvae affects their fitness, therefore reducing possible benefits from social facilitation [54].

#### Fruit type

The fruit type strongly influences the egg-clutch size and the outcome of larval development (Table 1). *D*. *suzukii* females lay more eggs on simply-shaped fruits (berries and drupes) than on fruits with a complex structure such as polydrupes (an assemblage of a high number of drupeoles), capsules (with internal physical partitioning as in *Euonymus europaeus*), pseudo- or composed fruits (like the rosehip of *Rosa canina* and pome fruits of *Pyrus calleryana*, *Mespilus germanica* and *Crataegus monogyna*). These complex fruits, more fibrous than the others, may hamper the migration of larvae within their flesh and reduce the efficiency of food intake by the larvae. Although the polydrupes of the *Rubus* species force the larvae to exit and then migrate to access other parts of the fruit, this physical barrier does not decrease the success of larval development (Table 1 and Figs 1 and 2). Many studies have shown the impact of fruit type on fruit attractiveness to frugivorous insects [53], including *Drosophila* species [113–115], although fruit discrimination is complex and depends on both the physical and chemical traits of the fruit [116]. The recent works on *D*. *suzukii* attractants focus almost exclusively on chemical components because they significantly increase our fundamental knowledge of the biology of the fly [108] and, predominantly, because the optimal chemical composition of bait is actively researched for the elaboration of drosophila traps used for fly monitoring and crop protection [117, 118]. Although the shape of bait is neglected in fly-trap design, our results show that the simply shaped structures of berries and drupes (oval or sphere) are preferred by *D*. *suzukii* females and could be drawn at the surface of plastic traps or used to pattern a new generation of solid baits containing chemical attractants.

#### Fruit colour

Fruit colour is an important visual trait for fruit flies’ oviposition behaviour [119, 120]. *D*. *suzukii* can lay eggs in fruits of various colours: black (*Atropa belladonna*, *Sambucus nigra*), blue (*Ribes sanguineum*), red (*Aucuba japonica*, *Arum maculatum*, *Fragaria vesca*, *Ribes rubrum*), pink and purple (*Gaultheria procumbens*, *Lonicera nitida*), orange (*Hippophae rhamnoides*), brown (*Pyrus calleryana* 'Chanticleer') or white (*Symphoricarpos albus*, *Viscum album*; Fig 2 and Appendix B). Therefore, the way *D*. *suzukii* uses colour to select elicited fruits remains a moot point and experimentation using coloured objects or host fruit mimics [51, 121] is still needed. The coloured area of the fruit could also be used by sexually mature flies as a rendezvous site for courtship and mating [121, 122]. Whatever the mechanisms governing fruit choice, *D*. *suzukii* tolerance to various fruit colours may be one key-factor explaining its polyphagy. Without this plasticity in the visual selection of the fruit, the fly would not have been able to infest such a large range of fruit. By using a great diversity of fruit colours, *D*. *suzukii* might also disorientate some potential parasitoids which could be attracted to a specific range of colours [123], even if kairomones also play an important role in host selection by parasitoids [124]. This plasticity in visual attractiveness could be a behavioural innovation accompanying the evolutionary changes in the fly’s morphology [29, 66].

The evolution of the fruit-penetrating ovipositor is a major morphological innovation that differentiates *D*. *suzukii* from its close relatives. The ability to drill holes into the skin of fresh fruits allows access to a new ecological niche [29]. This mutation in the fly’s ecology necessarily includes additional neurological, lifecycle, and physiological adaptations to find non-rotted fruits [66] and to identify their various colours.

#### Fruit skin

The first physical barrier developed by fruits against insects is their skin [39, 125]. *D*. *suzukii* females lay more eggs in rough-skinned fruits than in those showing a smooth, waxy, shiny or pruinose coating (Table 1). A waxy texture may indicate the presence of hydrophobic coatings and the presence of specific molecules [126] that may act as deterrents to insects. Pruinose surfaces contribute to the mechanical strength of plant tissues, to the cueing of host-pathogens/insects recognition, to the reduction of contamination and pathogen attacks, and to promoting or preventing insect attachment and locomotion [127–129]. This may contribute to explain why some pruinose fruits (*Viburnum tinus*, *Polygonatum multiflorum*, *Paris quadrifolia*, *Mahonia* x *media*, *Berberis julianae*) or shiny waxy fruits (*Ruscus aculeatus*, *Rosa canina*, *Berberis thunbergii*) have been less attacked by *D*. *suzukii*. Nevertheless, host attractiveness may also depend upon many additional factors [30, 31, 39] like, for example, soluble sugar content, pH or volatile compounds. Therefore, complementary experiments must be conducted for an accurate understanding of fruit attractiveness and suitability to *D*. *suzukii*.

The capacity of the fly to lay eggs in fruits protected by various skin textures (waxy, pruinose skins) partly relies on its saw-tooth ovipositor which bypasses the textural defence of the skin, and represents an evolutionary innovation [18] and a new weapon [130, 131] in the introduction areas as well as an advantage over other *Drosophila* species (see Atallah *et al*. 2014 [29] for the rapid evolution of that weapon). The evolution of that organ, together with the associated behaviour of fresh fruit recognition [18] provided *D*. *suzukii* with the ability to use the flesh of a large diversity of fresh fruits and to acquire these resources before other *Drosophila* species which mostly lay eggs on rotten fruits. As fresh fruits hanging on plants are neglected by other local *Drosophila* species (see *D*. *subobscura* feeding only on fruits fallen on the ground in Poyet *et al*. 2014 [24]), *D*. *suzukii* benefits from an empty niche [132], avoids major resident competitors and natural enemies [133], and may consequently increase its performance in the interactions network of its novel ecosystem [134]. Increased invasiveness coupled with the absence of *Drosophila* competitors is likely to have helped the fly to colonize a great diversity of habitats and reproduction/feeding substrates.

### Temporal continuity of host availability along the four seasons

Two recent studies [135, 136] examined the seasonal variations of *D*. *suzukii* populations and their relationship with the phenology of cultivated fruits, but the phenology of wild and ornamental fruits has not been studied satisfactorily yet. With its polyphagous behaviour, *D*. *suzukii* is likely to find alternative host plants in natural and urbanized systems throughout the year (see Fig 3). The number of host plant species fruiting between April and May is lower but many flowers (*Prunus spinosa*, *P*. *avium*, *R*. *rubrum*, *R*. *nigrum*, *Cornus sanguinea*, *Crataegus monogyna*…) producing nectar may compensate for the absence of fruit and help *D*. *suzukii* adults to survive until the summer [27]. Other plant species, which are resistant to the infestation by *D*. *suzukii*, produce a large amount of nectar in autumn (*Hedera helix*) and may contribute to *D*. *suzukii* overwintering. In winter, the fruits of several native and exotic host plants (*Viscum album*, *Symphoricarpos albus*, *Hippophae rhamnoides*) remain hanging on parent plants, especially along coastal areas where the oceanic climate protects fruits from frost. Therefore, one issue is still at stake: do *D*. *suzukii* populations migrate between ecosystems (from forests in autumn and winter to gardens in spring and summer, for example) and between regional areas, in order to find fruiting or flowering plants? Coastal areas with a warm climate, small temperature variations and many host plant varieties (such as *Hippophae rhamnoides* subsp. *rhamnoides*) may represent a continental climatic corridor for the dispersal of the fly.

### *Drosophila suzukii*, a new environmental filter among plant communities?

*Drosophila suzukii* may be a time bomb in natural plant communities. As numerous plants with thin-skinned fleshy fruit reproduce and disperse in temperate European landscapes and given the damages caused on fruits [137], significant impacts of *D*. *suzukii* invasion could be expected on the plant communities. However, according to recent advances in the role of frugivorous insects on the regeneration of host plant species [110], the magnitude and direction of the impact of *D*. *suzukii* on plant species fitness remains uncertain. Indeed, frugivorous insects are not always pests [110] and they may produce negative, neutral or beneficial effects on plants according to their hosts’ autecology.

Phytophagous, and especially frugivorous, insects were first considered to be particularly damaging to hosts because they pre-empt the plants’ reproductive tissues [138], accelerate fruit decay, cause premature abscission of infested fruits [139] containing immature seeds, reduce fruit attractiveness for vertebrate endozoochorous dispersers (animals that disperse seeds via the ingestion of fruits, such as birds or mammals) [140, 141], which consequently increases competition between parent plants and offspring growing under their canopy. They are also major drivers for the evolution of host plant defensive traits [142]. Seed transport by animal dispersers is the main way for many plants to reproduce, colonize new habitats, and survive in the landscape matrix [143, 144]. Fruit removal and seed arrival in different habitats are processes related to frugivorous species [144, 145]. Among frugivorous vertebrates, birds are major vectors for fleshy fruits in Europe. They are highly sensitive to fruit quality and they discriminate and significantly reject fruits that have been attacked by insects [140, 141, 146, 147]. The alteration of the dispersal service provided by avian seed dispersers is known to cause plant regeneration collapses [144] and to produce cascading effects on ecosystem services [148]. As the fruits of 50% of the tested plant species can be damaged by *D*. *suzukii*, a high number of species could lose their ability to disperse and regenerate successfully. Even if the species damaged by *D*. *suzukii* are not economically important, they contribute to the equilibrium and services of the ecosystem. For instance, *Prunus avium* is used for wood production and many fruits from wild plants (*Rubus fruticosus*, *R*. *idaeus*, *Sambucus nigra*, *Fragaria vesca*, *Prunus spinosa* or *Hippophae rhamnoides*) are harvested for cooking and jam preparation or medicinal use, and marketed. By damaging ripening fruits, *D*. *suzukii* may modify the fertility and the dispersal performances of plant species, and may consequently change their frequency and place in the communities. A shift in the functional composition of forest communities could then occur: fleshy-fruited species infested by the fly would be disadvantaged to the benefit of plants reproducing through vegetative organs (stolons) or dry fruits/seeds (achenes, wing-bearing samaras, for example) not attacked by *D*. *suzukii* larvae. Consequently, anemochorous (seed dispersal by wind) and ectozoochorous (external transport of seeds by animals, in their fur for example) processes would be more likely to determine the patterns of species distribution than endozoochorous processes in the future ecosystems invaded by *D*. *suzukii*.

Several studies showed the neutral or positive effects of frugivorous insects on plant fitness [110, 142]. Indeed, the viability of embryos and the production of seeds are not necessarily affected by insect activity. By perforating the seed coat, removing the fruit pulp or accelerating fruit decay, insects may even stimulate germination, increase seed viability or attract frugivorous birds and mammals responsible for seed dispersal [110, 149, 150]. Regarding these contradictory plant-insect interactions and the complexity of the triad fruits—insects—frugivorous vertebrate dispersers [151], it seems difficult to predict the effect of *D*. *suzukii* on plant regeneration accurately. However, by pre-empting the organic matter contained in the fruit flesh of many plant species, *D*. *suzukii* will change the fluxes of matter and energy and the interactions in trophic networks and will become a new, non-negligible component of the ecosystem.

### A perspective in the ecological control of *Drosophila suzukii*

Although half the wild and ornamental plants with fleshy fruits may be considered to be reservoirs for the fly, some of the tested plants (*Pyracantha*, *Cotoneaster*…) attractive to *D*. *suzukii* gravid females are characterized by low success or absence of imago emergence (see Figs 1 and 2). These “trap plants” may lure the fly as they induce oviposition but do not allow the full development of larvae. Further investigations are needed to understand why larvae do not reach the adult stage in these fruits (presence of toxic compounds, low water and nutrient availability, fibrous structure…). These potential trap plants could be planted near sensitive crops (strawberries, grapes) to reduce the amount of pesticides. Choice tests between potential wild (and non invasive) trap-plants and agronomic ones will be the next step towards the ecological control of the invasive fly.
